# Supplementary material for: Inhibition of Transglutaminase 2 as a Therapeutic Strategy in Celiac Disease—In Vitro Studies in Intestinal Cells and Duodenal Biopsies
Source: Int J Mol Sci. 2023 Mar 1;24(5):4795. doi: 10.3390/ijms24054795 (PMC10002517; doi:10.3390/ijms24054795)
Supplement: Supplementary file 1 [file ijms-24-04795-s001.zip › ijms-2164290-supplementary.pdf]

Supplemental 1: Processing of confocal images of CD biopsies

For each patient, five representative images were obtained. Settings of the laser, the photomultiplier and the acquisition modes (Pixel dell time 2.4, , 1024 pixels, pinhole 30 μm) were equally applied in all images. The following macro for thresholding of the intestinal lamina propria was used:

```
run("Duplicate...", " ");
run("Grays");
run("Enhance Contrast...", "saturated=0.01 normalize");
run("Minimum...", "radius=1");
run("Subtract Background...", "rolling=50 sliding");
setOption("ScaleConversions", true);
run("8-bit");
run("Auto Threshold", "method=Moments white");
run("Create Selection");
roiManager("Add")
```
